# From a Basic Microalga and an Acetic Acid Bacterium Cellulose Producer to a Living Symbiotic Biofilm

**DOI:** 10.3390/ma12142275

**Published:** 2019-07-15

**Authors:** Vítor Nóbrega, Marisa Faria, Antera Quintana, Manfred Kaufmann, Artur Ferreira, Nereida Cordeiro

**Affiliations:** 1 LB3, Faculty of Science and Engineering, University of Madeira, 9000-390 Funchal, Portugal; 2 Oceanic Observatory of Madeira (OOM), ARDITI, Madeira Tecnopolo, 9020-105 Funchal, Portugal; 3 Banco Español de Algas, Instituto de Oceanografía y Cambio Global (IOCAG), Universidad de Las Palmas de Gran Canaria, 35214 Telde, Spain; 4 Marine Biology Station of Funchal, Faculty of Life Sciences, University of Madeira, 9000-107 Funchal, Portugal; 5 CIIMAR-Interdisciplinary Centre of Marine and Environmental Research, University of Porto, 4450-208 Matosinhos, Portugal; 6 CICECO, Aveiro Institute of Materials, University of Aveiro, 3810-193 Aveiro, Portugal

**Keywords:** bacterial cellulose, microalgae, living biofilm, *Chlamydomonas debaryana*, *Komagataeibacter saccharivorans*, symbioses

## Abstract

Bacterial cellulose (BC) has recently been the subject of a considerable amount of research, not only for its environmentally friendly biosynthesis, but also for its high potential in areas such as biomedicine or biomaterials. A symbiotic relationship between a photosynthetic microalga, *Chlamydomonas debaryana*, and a cellulose producer bacterium, *Komagataeibacter saccharivorans*, was established in order to obtain a viable and active biofilm. The effect of the growth media composition ratio on the produced living material was investigated, as well as the microalgae biomass quantity, temperature, and incubation time. The optimal temperature for higher symbiotic biofilm production was 30 °C with an incubation period of 14 days. The high microalgae presence, 0.75% *w/v*, and 60:40 HS:BG-11 medium (*v/v*) induced a biofilm microalgae incorporation rate of 85%. The obtained results report, for the first time, a successful symbiotic interaction developed in situ between an alkaline photosynthetic microalga and an acetic acid bacterium. These results are promising and open a new window to BC living biofilm applications in medical fields that have not yet been explored.

## 1. Introduction

Bacterial cellulose (BC), an exopolymer of *Gluconacetobacter* sp. bacteria, a subgenus of *Acetobacter*, has many unique properties such as a sophisticated 3D porous network, long fibre, high crystallinity, high water absorption, and retention capacity [1,2,3]. The BC chemical structure is composed by (1→4)-D-anhydroglucopyranose chains bounded through *β*-glycosidic linkages. BC’s geometry is determined by the intra-molecular and inter-molecular hydrogen-bonding network, hydrophobic and van der Waals interactions, and forms parallel chains (Cellulose Type I) [2]. Despite similarities to other nanocellulose types like cellulose nanofibers (CNF) or cellulose nanocrystals (CNC), BC nanofibrils have unique properties such as more crystallinity, a higher degree of polymerization, and the ability to form an ultrafine web structure that makes it difficult to disperse [4]. Furthermore, BC also has a good mechanical strength and allows for a relatively easy shaping control, e.g., through a container´s shape where the bacteria are cultured [5]. This biopolymer is generally produced in a static media culture and forms a gelatinous membrane on the interface between air and the culture (aerobic microorganisms) [6]. Moreover, an important asset of BC is its excellent biocompatibility. Many studies have described the successful interactions between BC and other cell types or microorganisms. One example is described by Hu et al. who reported that there was a positive biocompatibility with human osteoblasts, where the osteoblasts were able to grow and rapidly attach themselves in the BC [7]. Furthermore, Czaja et al. reported a new wound healing system based on BC, which clinically investigated the treatment of skin burns on humans [8]. In this research, the wound healing effects of never-dried BC membranes were compared with conventional gauze wound dressings (controls). These studies were proceeded by in vivo tests conducted on animal models and showed that BC membranes were fully biocompatible and do successfully protect burn wounds by preventing excessive external fluid loss, which stimulates a more rapid healing process.

In order to create the optimal conditions for the mutual growth and a sustainable symbiotic process (association between two or more organisms), some changes must be made to each component of this process, for example to generate an environment that can endure two or more different metabolisms and their conditions [9,10]. One example of well-studied mutuality relationships are lichens, which are commonly known as a symbiotic association of a fungus and a partner containing chlorophylls, either cyanobacteria, green algae, or both [11]. Chlorophyll enriched partners provide photosynthetically fixed carbon as the source of energy for the system, while fungus promotes a suitable substrate for both. However, some literature also reports that lichens produce antibacterial and antifungal compounds [12]. Moreover, recently, many research reports suggest that there is a symbiosis phenomenon between lichens and microorganisms, such as bacteria [13]. These observations unlock a whole new range of possibilities by showing that there could be a conceivable mutual relationship between photosynthetic organisms and microorganisms like bacteria. In fact, a few cases have already been reported. One example is the mutualistic relationship between *Chlorella vulgaris* (photosynthetic green microalgae) and a symbiotic bacterium, *Pseudomonas* sp. [14]. Guo and Tong reported different interactions between algae and bacteria when different incubation conditions were applied. Moreover, adaptability is a major factor that contributes to the establishment of a symbiotic relationship. Das et al. reported a symbiosis between *Acetobacter aceti* and *Chlamydomonas reinhardtii* were cultured simultaneously [15]. The symbiotic conditions were described as acidic (pH below 6.5), with a 70:30 ratio between the bacteria medium (glucose and yeast) and the microalgae medium (tris-acetate-phosphate), with an optimal temperature between 28 and 30 °C. In addition, a symbiotic growth by an acetic acid bacterium and an acid microalga has already been reported [15,16]. However, literature is still lacking on a successful high pH symbiosis achievement. An alkaline association could enable this kind of bacteria to produce a 3D network BC for incorporation of alkaline microalgae such as *Chlamydomonas debaryana* (a model microalga), and thus, this could define whole new horizons in biomedical treatments and technology.

Furthermore, microalgae are also able to use light energy and convert it throughout several membrane protein complexes into electrochemical energy, which is then used to assimilate CO_2_. This process releases oxygen, an essential molecule for energy production in aerobic organisms such as *Komagataeibacter saccharivorans* [17].

The aim of this study was to obtain, for the first time, a biofilm composed by *C. debaryana* cells (basic microalgae) and a BC 3D network produced by *K. saccharivorans* (acid bacterium), considering its biocompatible and high-water absorption properties. The optimal composition of the culture media which supports symbiotic growth of the living biofilm, the viability of the integrated microalgae, variations on medium acidity and incorporation ratios were then studied. The obtained biofilm was characterized morphologically and structurally through ATR-FTIR, SEM, and fluorescence microscopy methods.

## 2. Results and Discussion

### 2.1. Growth Symbiotic Media Optimization

#### 2.1.1. Carbon Source

The production of biomaterials by microorganisms is a process that requires a huge amount of energy, however, its production also has many different functions and benefits. For example, BC has many functions to bacteria, such as the acquisition of oxygen, the prevention of ultraviolet radiation damage, the enhancement of antibiotic resistance, and the retention of moisture [18,19]. Furthemore, BC production process of *Gluconacetobacter* strains is already well documented and indicates that glucose is the main precursor for cellulose synthesis [20]. Thus, the compounds capable of being transformed into glucose are theoretically viable carbon sources for cellulose producing bacteria. However, an efficient BC production is highly dependent on the carbon source selection [19]. Bacteria can use several sugars for metabolic purposes through distinct pathways. This ability induces the production of different metabolic products that are extruded from bacteria and influences the medium pH value [21].

Previous pilot studies have shown that the acidity of *K. saccharivorans* culture media was in a hostile environment for *C. debaryana*, which consequently resulted in an inhibition of the population growth or in a significant decrease of the initial cell population. Therefore, a reformulation of the HS culture medium was necessary for the microalgae’s survival. Based on this, different carbon sources in the culture media were studied. Table 1 displays the culture media’s final pH values for the three concentrations of the three different carbon sources. The results show that replacing a carbon source influences the culture’s pH *K. saccharivorans* culture with the use of glucose produces a significantly (*p* < 0.05) more acidic environment (Table 1). This can be explained by the bacteria induced production of (keto) gluconic acids through an oxidative process, which lowers media pH [22,23]. Using the HS medium with fructose as a carbon source induces a fairly neutral pH setting through *K. saccharivorans bacterial* metabolism (Table 1). Therefore, through performing an analysis on fructose metabolism from *Gluconacetobacter* genera on KEGG Database, fructose can be transformed into fructose-6P, which is a precursor of many other metabolites involved in the amino sugar and nucleotide sugar metabolism [24]. These metabolisms promote alkaline environments due to the ammonia groups’ presence in several metabolized molecules, their possible catabolism and may explain fructose-based HS medium final pH [17].

Sucrose, as a disaccharide, has a more complex process before it matches *K. saccharivorans* metabolic requirements. Since its breakdown must first occur for it to be used as a carbon source [25]. Sucrose breakdown by *K. saccharivorans* occurs in an intramembrane protein known as levansucrase and it results in two monomers–fructose and glucose [26,27,28]. In normal circumstances, the availability of these two sugars results in bacteria metabolism producing an increase in the medium acidity. However, sucrose-based HS media (1, 2, or 4 wt %) did not cause a final medium acidification through bacteria metabolism, but instead induced the process of alkalization. This phenomenon has been previously documented, but it has never been recorded with such an alkaline pH value as it was in this study [29].

In order to better understand the chemical behaviour of the carbon source metabolization and its associated metabolites, a carbohydrate’s analysis using HPLC was employed. An increment of gluconate was observed as a result of the extended incubation time. This gluconate increment was seen to have influence on culture media pH value, since it rose from 3.25 (initial incubation—0 day) to 8.4 (7 days after incubation). Gluconate is a gluconic acid salt, which has an alkaline profile in water solution with a pH value that ranges between seven and eight.

Bacteria tend to use glucose as the preferential carbon source and produce (keto) gluconic acid [23]. In the glucose deficiency, bacteria have the ability to metabolize (keto) gluconic acid and produce gluconate, which results in a pH increase [30]. This can explain the pH increase found in this research.

Fructose, on the other hand, may be used for other metabolic purposes such as the ones previously cited (amino sugar and nucleotide sugar metabolism). This same pattern may not occur, when there is only fructose available in the medium, because the bacteria may transform all the sugar available through a central pathway for its energy production.

Since a higher pH value was obtained in the HS medium with sucrose, and *C. debaryana* does not grow in low pH medium due to its alkaline profile, this medium (1 wt %) was used to study *K. saccharivorans* in all the symbiotic environments.

#### 2.1.2. Symbiotic Media Constitution

**Figure 1:**
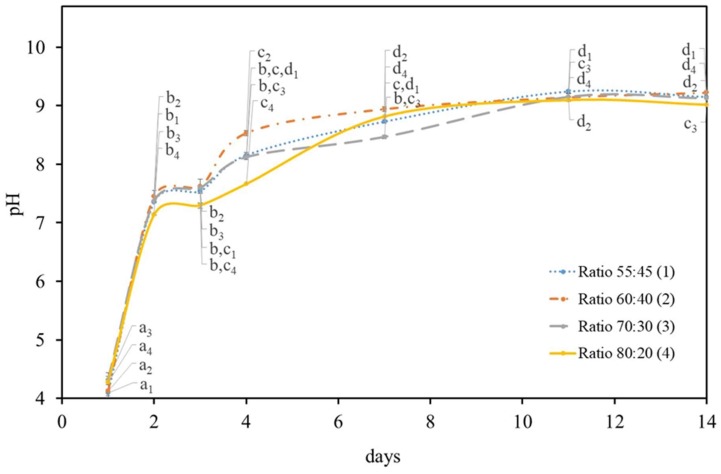
pH variation in the different HS:BG-11 ratio media during the 14 days of incubation of *K. saccharivorans* at 30 °C. Values in the same curve that do not share a common letter (index according to each HS:BG-11 ratio) are statistically different (*p* < 0.05).

After carbon source optimization took place, the biofilm production by *K. saccharivorans* was studied in different HS:BG-11 culture medium (symbiotic medium) ratios at 7 and 14 days (Figure 1). To understand if the BG-11 medium nutrients would affect *K. saccharivorans*’s ability to perform a medium alkalinisation, the pH level was recorded at various times in order to monitor the change in the acidity of the culture media. The initial pH for all the medium ratios was acid (4.2 ± 0.3). Lastly, the alkalization process started to take place after two days of incubation with a quick increase of (72%) in the pH level followed by a steady increase from the 7th until the 14th day.

The bacteria presented a similar pH variation in all the HS:BG-11 symbiotic media ratios during the experiment, with a rapid increase on the 4th day (Figure 1). After the 7th day, the pH values of each ratio maintained a similar (*p* < 0.05) value and were seen to be analogous to the 7th day pH value of the sucrose-based HS medium trials (Table 1). Up until the 14th day, they showed a minor increment (3.4%) reaching a pH value of 8.9 ± 0.01.

The statistical analysis was performed for each day in each of the tested symbiotic media ratio. It found that the pH values of 60:40 symbiotic media ratio were statistically different on the first three days, which reveals a well individualized increase of pH and a stabilization (from the 7th day) to a pH value adjacent to 9.

**Figure 2:**
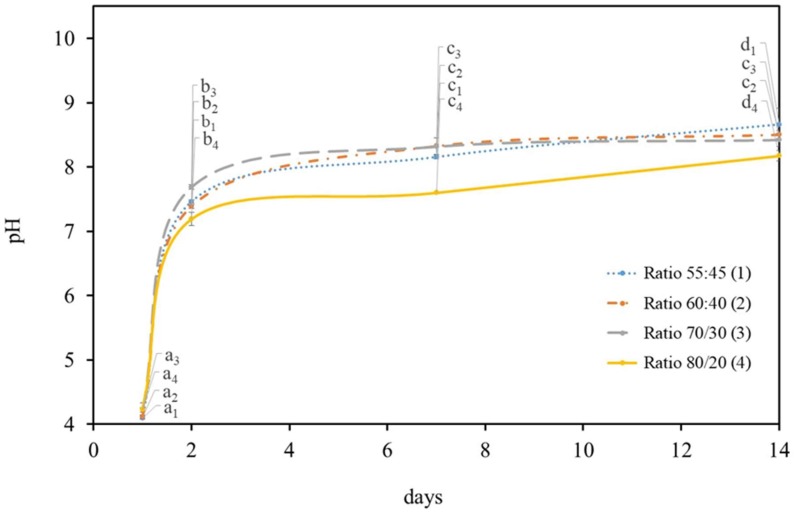
pH variation in different HS:BG-11 ratios media, for 14 days of *K. saccharivorans* and *C. debaryana* (0.75% (*w/v*)) at 30 °C. Values in the same curve do not share a common letter (index according to each HS:BG-11 ratio) are statistically different (*p* < 0.05).

Previous pilot experiments have shown that the addition of both microorganisms at the initial acidity (day 0) can be seen to have a negative effect on the microalgae survival under all HS:BG-11 ratios tested. However, on the 2nd day a large pH increase was observed (7.44 ± 0.03), which was very close to the regular microalgae media pH (7.4) and suitable to support microalgae metabolism and growth. Based on this, the microalgae were added after the bacteria´s two-days incubation to the symbiotic medium. In these conditions, the pH was recorded for several days to assess both the microorganisms’ interaction and to understand *C. debaryana*’s role in the medium alkalization (Figure 2). It was observed that the variation in the pH of media was similar (*p* < 0.05) to the one found previously without microalgae under the same conditions. Thus, the presence of microalgae does not induce a significant change in the pH media.

### 2.2. Growth Temperature

Another important factor studied was the optimum temperature of symbiotic culture. Biofilm production was assessed under two temperatures, 25 and 30 °C. The temperature selection considered optimal growth temperatures of each microorganism, *C. debaryana* (25 °C) and *K. saccharivorans* (30 °C). Both these temperatures were tested in periods of 7 and 14 days with all symbiotic ratios. Alongside the temperature assessment, the incorporation rates of formed-biofilms were also recorded.

**Figure 3:**
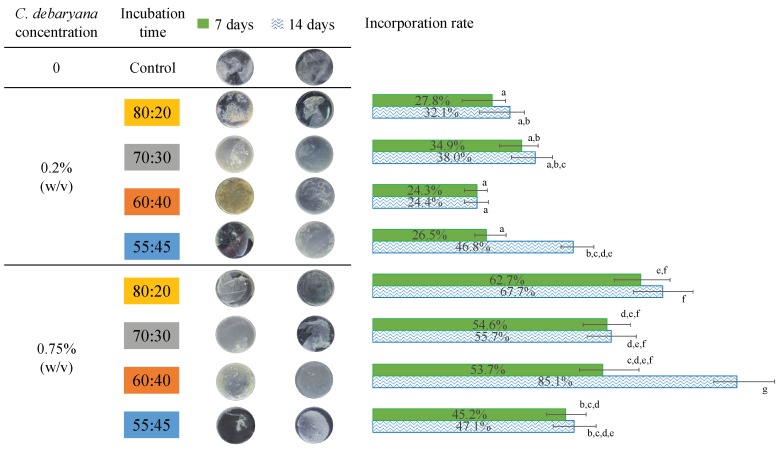
Symbiotic biofilms obtained to the different HS:BG-11 media ratios during 7 and 14 incubation days, and the *C. debaryana* incorporation rate. Values in the same column that do not share a common superscript are significantly different (*p* < 0.05).

*K. saccharivorans* under 25 °C, during a 7 days’ period and a 14 days’ period, on all the tested symbiotic ratios, produced small particles of cellulose, and in some cases, BC entangled a few microalgae. Taking this into consideration, the same methodology was applied but under 30 °C. At this temperature, all the symbiotic media ratios enabled the symbiotic biofilm production, even after seven days of incubation (Figure 3). Thus, one can conclude that the temperature increment was beneficial to the bacteria exopolymer extrusion.

**Figure 4:**
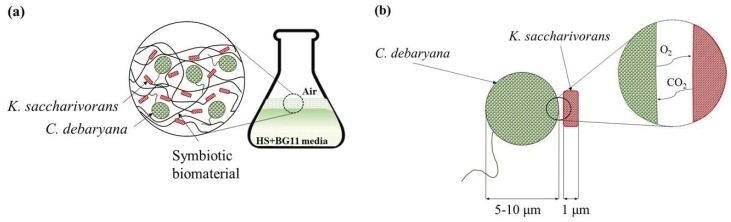
Illustration of (**a**) symbiotic biofilm formation by *K. saccharivorans* and *C. debaryana* in HS:BG-11 culture media and (**b**) CO_2_ and O_2_ transfer between *C. debaryana* and *K. saccharivorans*.

To study the effect of the *C. debaryana* amount on the symbiotic biofilms formed, two different concentrations were studied: 0.2 and 0.75% (*w/v*). The higher *C. debaryana* presence (0.75% (*w/v*)) assisted the production of living symbiotic biofilms with an improved consistency and rounded shape. An explanation for this is that the higher the presence of microalgae induced a raise in the O_2_ media content (Figure 4). As *K. saccharivorans* is an aerobic microorganism, the higher O_2_ media content stimulates its metabolic pathways, resulting in a more active production of exopolymers (cellulose fibres). This superior active extrusion allows for a more physical space for the microalgae enrolment into the biofilm during its formation and causes significantly higher microalgae incorporation rates (Figure 3). The highest (*p* < 0.05) biofilm incorporation rate (85.1%) was recorded under 30 °C, with a 14 day long incubation, a *C. debaryana* presence of 0.75% (*w/v*) and on a 60:40 (HS:BG-11) symbiotic media ratio.

### 2.3. Biofilm Characterization

**Figure 5:**
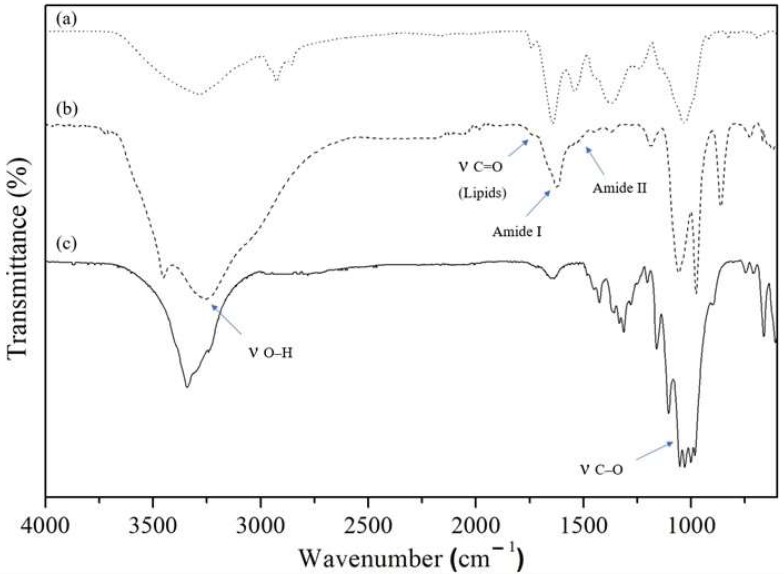
ATR-FTIR spectra of (**a**) microalgae *C. debaryana*, (**b**) biofilm produced by symbiotic growth of *K. saccharivorans* and *C. debaryana* and (**c**) regular BC produced by *K. saccharivorans*.

ATR-FTIR spectra of biofilm shows typical bands for the cellulose based materials (Figure 5), such as 3300 cm^−1^, corresponding to stretching O-H bond; 2990 cm^−1^, corresponding to stretching C-H bond; and 1050 cm^−1^ corresponding to stretching C-O bond [23]. The microalgae presence can be assessed by looking for macromolecules’ distribution pools, such as lipids and proteins pools. According to Giordano et al., the lipid band (stretching band C=O bond) is located around 1740 cm^−1^ and amides I/II bands (representing proteins) are located around 1660 cm^−1^ and 1540 cm^−1^, respectively [31]. All three bands are absent in regular BC (Figure 5c) spectra, but present in biofilm ATR-FTIR spectra (Figure 5b).

The ATR-FTIR spectra were used to determine the ratio of cellulose *Iα* and *Iβ* allomorphs [32]. The *Iα/Iβ* ratio was found to be 1.01 and 0.99 for regular BC and symbiotic biofilm. Both *Iα/Iβ* ratios are higher than reported for the bacterial cellulose due to the sugar source and the higher temperature used in these experiments (30 °C), which decreases the *Iα* level [33,34]. However, the similarity between *Iα/Iβ* ratios indicates that the microalgae incorporation didn’t induce any changes in the *Iα/Iβ* ratio. Therefore, microalgae presence did not influence the normal *K. saccharivorans* cellulose production of equivalent *α* and *β* cellulose types.

**Figure 6:**
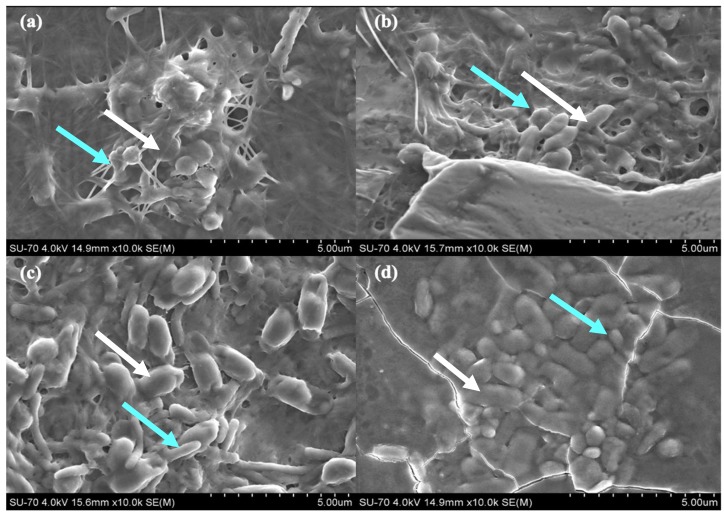
SEM micrograph of symbiotic biofilms produced by bacteria *K. saccharivorans* (blue arrow) and microalgae *C. debaryana* presence (white arrow), at 30 °C after 14 day incubation, in different HS:BG-11 symbiotic media ratio: (**a**) 55:45; (**b**) 60:40; (**c**) 70:30; and (**d**) 80:20.

The living symbiotic biofilms from each ratio HS:BG-11 of symbiotic media were observed by SEM (Figure 6). Biofilm’s micrographs show the presence of both microorganisms and the synthetized cellulose fibres network: Rod-shaped cells are *K. saccharivorans* and the oval shaped cells are *C. debaryana*. For this reason, it can be seen that the morphology of the cellulose network of the different samples is similar.

**Figure 7:**
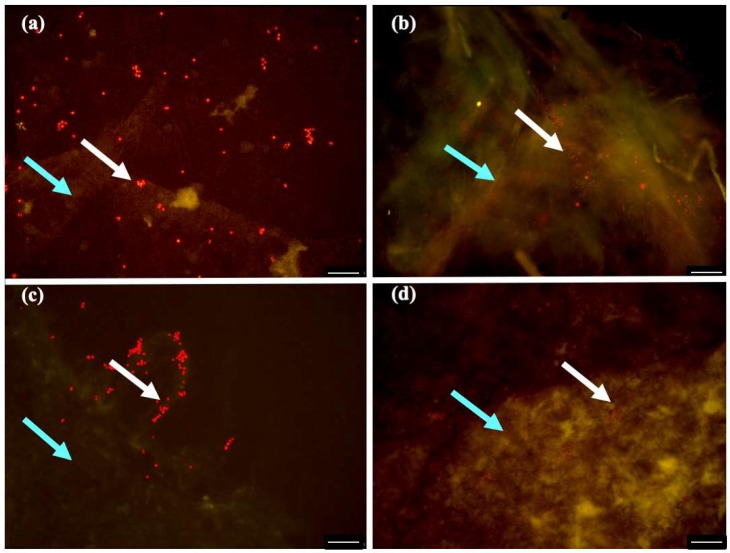
Fluorescence micrographs showing the viability of *C. debaryana* cells (white arrow) immobilized in biofilms (blue arrow) produced at 30 °C and after 14 day of incubation, in different HS:BG-11symbiotic media ratios: (**a**) 55:45; (**b**) 60:40; (**c**) 70:30 and (**d**) 80:20. Scale bar, 100 μm.

As photochemically active chlorophylls are a requirement for photosynthesis, their presence proves that the microalgae cells were alive. Fluorescent microscopy was used to assess the microalgae cells viability (Figure 7). The signal intensity indicates that functional chloroplasts were present. Furthermore, within these chloroplasts, there are chlorophylls, which are capable of being excited and can emit radiation, which is typical for living photosynthetic organisms [17]. Therefore, all the symbiotic biofilms obtained, contain viable and metabolic active *C. debaryana* cells. Nonetheless, the biofilm produced in 55:45 (HS:BG-11) ratio symbiotic media under 30 °C and after 14 days of incubation presented the widest microalgae distribution (Figure 7a). The obtained biofilm in a 60:40 (HS:BG-11) ratio (better incorporation ratio) also revealed a wide distribution of microalgae cells, however, cellulose density and reticulation resulted in a more compact setting of these cells (Figure 7b).

## 3. Materials and Methods

### 3.1. Microorganisms and Grown Medium Conditions

The cellulose producing bacterium employed was *K. saccharivorans* isolated from vinegar, and was grown in Hestrin-Schramm (HS) medium (20 g/L D(+)-glucose, 5 g/L yeast extract, 5 g/L peptone, 2.7 g/L Na_2_HPO_4_, 1.15 g/L citric acid, 5 ml/L ethanol, and 2 ml/L acetic acid). The erlenmeyer flasks containing bacteria and HS medium were placed in an incubator (MM Group Incucell, Munich, Germany) at 30 °C. Here the HS medium optimization was produced changing the carbon source and its concentration and fructose, sucrose and glucose, at concentrations of 1, 2, and 4 wt %, were selected as carbon sources separately. The pH for all the HS media was adjusted with acetic acid to 3.25 ± 0.01.

The *C. debaryana* (BEA0067) was kindly provided by the Spanish Bank of Algae (BEA, Gran Canaria, Spain). The cells were grown (photoautotrophically) in a BG11 medium (deionized water), and were cultured in an Erlenmeyer flask at pH of 7.4 and later placed in an incubator at 25 ± 1 °C without agitation.

The setup was illuminated using three 18 W cool white lights (3500 Lux) that provided a photon flux of 47.25 µmol m^−2^ s^−1^. The cells were grown for three days and harvested before their late logarithmic growth phase.

Lastly, all the materials used for cell growth process were previously autoclaved at 121 °C for 15 min.

### 3.2. Symbiotic Process

The microorganisms were grown individually and in a symbiosis in a mixture of HS and BG11 culture media. Symbiotic media ratio was designed to sustain both microorganisms at 80:20, 70:30, 60:40 and 55:45 of HS:BG-11 media (*v/v*). Incubation was then performed at 25 ± 1 °C or 30 ± 1 °C, during 7 or 14 days. The ratios experiments were performed based on an inoculum of *K. saccharivorans* with an optical density of 0.6 at 600 nm and based on two *C. debaryana* percentages, 0.2% and 0.75% (*w/v*). Lastly, the produced biofilms were conserved under fresh symbiotic media and respective conditions.

### 3.3. High Performance Liquid Chromatography (HPLC)

HPLC analysis was performed in a Shimadzu Nexera X2 system equipped with a LC-30AD pump, a SIL-30AC autosampler, a DGU-20A (SR) degasser, a CTO-20AC column oven and coupled to a RID-20A refractive index detector. A Merck Polyspher CH-Pb column (300 × 7.8 mm; ref: ref 51278) was used at a constant oven temperature of 80 °C with an isocratic flow of water with a flow rate of 0.6 mL min^−1^. The injection volume for each sample was 50 µL. Sucrose, fructose, glucose, and sodium gluconate were used as carbohydrates standards.

### 3.4. Determination of Incorporation Rate

The harvesting efficiency was calculated using the following Equation (1):

Incorporation rate (%) = (1 − *R*/*O*) × 100% (1)

where *R* is the residual concentration of microalgal cells in the culture media after harvesting, and *O* is the original concentration of microalgal cells. The concentration of microalgal cells was determined using a hemocytometer (Neubauer-improved, Marienfeld, Germany) with an optical microscope (Olympus BX41, Tokyo, Japan).

### 3.5. Attenuated Total Reflection Fourier Transform Infrared Spectroscopy (ATR-FTIR) Analysis

ATR-FTIR spectra were obtained using a Perkin-Elmer (Waltham operating at 4.0 kV, in the field emission mode. The films were deposited on a steel plate and coated with carbon before analysis using an EMITECH K950X Turbo Evaporator (Montigny-le-Bretonneux, France).

### 3.6. Scanning Electron Microscopy (SEM) Analysis

SEM analysis of biofilms were subjected to a pre-treatment where the synthetized biofilms were washed three times with water, at 2000 rpm for 10 min, in order to remove the cells which were not integrated and the culture media compounds. Subsequently, they were fixed using 2.5% glutaraldehyde in 0.1 M sodium phosphate buffer at pH 7.2 and kept overnight at a temperature of 4 °C. Afterwards, they were washed two times with a 0.1 M sodium phosphate buffer at pH 7.2, and then dehydrated with ethanol (30, 50, 70, 90 and 100% (v/v)) before they were freeze-dried. The samples were then preserved in a desiccator until analysis.

SEM images were acquired with a HR-FESEM SU-70 Hitachi microscope (Tokyo, Japan) operating at 4.0 kV, in the field emission mode. The films were deposited on a steel plate and coated with carbon before analysis using an EMITECH K950X Turbo Evaporator (Montigny-le-Bretonneux, France).

### 3.7. Fluorescence Microscopic Analysis

The microalgae viability was assessed using fluorescence microscopy (Leica DM2700 P, Wetzlar, Germany). Taking into consideration the presence of the chlorophylls in *C. debaryana*, an excitation radiation with a wavelength between 450 and 490 nm was applied. The fluorescence was then observed at an emission radiation of 515 nm.

### 3.8. Statistical Analysis

The statistical analysis of the data was carried out by using the IBM SPSS Statistics V25 software. The differences in the measurements of a given parameter were assessed by one-way analysis of variance (ANOVA), followed by a Tukey’s post hoc analysis.

## 4. Conclusions

This work describes a symbiotic relationship between an acetic acid bacterium, *K. saccharivorans*, and an alkaline microalga, *C. debaryana*, for a new living symbiotic biofilm production. In this study it was found that the *K. saccharivorans* can form cellulose films under alkaline conditions, using sucrose as a carbon source and making the symbiotic relationship with photosynthetic microalga *C. debaryana* possible. The microalgae immobilisation took place in actual time along with the BC biofilm production. The microalgae added on the 2nd day of the bacteria incubation, with the temperature of 30 °C and a ratio 60:40 of HS:BG-11 symbiotic medium was found to be the most affective condition for the microorganisms’ growth, the living biofilm production and the microalgae incorporation into the BC network. This procedure can be used in applications in both medical and biological fields; such as in the development of living biofilms for the medical treatment of wounds, where oxygen plays an important role as an angiogenic factor. Additionally, it is also for the establishment of new biological tissues, and the symbiotic relationships of two or more microorganisms.

## Figures and Tables

**Table 1:** Final pH value of growth HS media using different carbon sources, after seven days of *K. saccharivorans* incubation. Initial pH of 3.4 ± 0.01 for all the media.

Carbon Source	wt %	pH Final
Glucose	1	3.3 ± 0.09 ^a^
	2	2.9 ± 0.01 ^a^
	4	3.0 ± 0.01 ^a^
Fructose	1	5.4 ± 0.16 ^b^
	2	7.2 ± 0.02 ^c^
	4	7.1 ±0.19 ^c^
Sucrose	1	8.4 ± 0.08 ^d^
	2	8.3 ± 0.01 ^d^
	4	8.2 ± 0.10 ^d^

Values in the same column not sharing a common superscript (a, b, c, d) are statistically different (*p* < 0.05).
